# The role of gut archaea in the pig gut microbiome: a mini-review

**DOI:** 10.3389/fmicb.2023.1284603

**Published:** 2023-10-09

**Authors:** Jianbo Yang, Routing Chen, Yunjuan Peng, Jianmin Chai, Ying Li, Feilong Deng

**Affiliations:** ^1^Guangdong Provincial Key Laboratory of Animal Molecular Design and Precise Breeding, College of Life Science and Engineering, Foshan University, Foshan, China; ^2^School of Life Science and Engineering, Foshan University, Foshan, Guangdong, China; ^3^State Key Laboratory of Swine and Poultry Breeding Industry, Guangzhou, China

**Keywords:** pig gut microbiota, gut archaea, probiotics, pig, feed efficiency

## Abstract

The gastrointestinal microbiota of swine harbors an essential but often overlooked component: the gut archaea. These enigmatic microorganisms play pivotal roles in swine growth, health, and yield quality. Recent insights indicate that the diversity of gut archaea is influenced by various factors including breed, age, and diet. Such factors orchestrate the metabolic interactions within the porcine gastrointestinal environment. Through symbiotic relationships with bacteria, these archaea modulate the host’s energy metabolism and digestive processes. Contemporary research elucidates a strong association between the abundance of these archaea and economically significant traits in swine. This review elucidates the multifaceted roles of gut archaea in swine and underscores the imperative for strategic interventions to modulate their population and functionality. By exploring the probiotic potential of gut archaea, we envisage novel avenues to enhance swine growth, health, and product excellence. By spotlighting this crucial, yet under-investigated, facet of the swine gut microbiome, we aim to galvanize further scientific exploration into harnessing their myriad benefits.

## 1. Introduction

The gastrointestinal tract (GIT) of pigs, similar to other livestock, hosts a complex and dynamic microbial community consisting of bacteria, fungi, viruses, and archaea, which plays a crucial role in enhancing host health and performance (Upadhaya and Kim, 2022; Vasquez et al., 2022; Zhao et al., 2023; Zhuang et al., 2023). The gut microbiota in pigs mediates nutrient metabolism, modulates the immune system, and provides colonization resistance against pathogens, functioning as a key determinant in the symbiotic relationship between the pig host and its microbial inhabitants. In recent years, numerous studies have examined the influence of the gut microbiome on the health (De Vries and Smidt, 2020; Duarte and Kim, 2022; Deng et al., 2023) and economically significant traits of pigs, identifying several key bacteria and potential probiotics that affect pig growth performance (Giang et al., 2011; Wang et al., 2019; Lee et al., 2022). In summary, research on the interactions among the complex microbiota residing in the pig gastrointestinal tract, as well as the interplay between bacteria and host, is proving valuable for improving pig farming efficiency.

Archaea are prokaryotic microorganisms that are distinct from eukaryotes and bacteria, and are commonly found in the bodies of human and animals (Matarazzo et al., 2012; Bang and Schmitz, 2015). While archaea share morphological features with bacteria, they have developed unique biochemical and metabolic characteristics (Pace, 1997; Schleifer, 2009), such as thermophily and heat resistance, during their long evolutionary history (Macario et al., 1999; Baker et al., 2020). In humans, archaea colonize the infant gut at birth and interact with bacteria, viruses, and the human body, thereby exerting an influence on health (Wampach et al., 2017; Korpela and de Vos, 2018). Trimethylamine N-oxide (TMAO) is a molecule produced from choline, betaine, and carnitine through gut microbial metabolism. Elevated levels of TMAO have been associated with cardiovascular risks (Janeiro et al., 2018). Studies suggest that intestinal archaea may contribute to obesity and reduce TMAO-related diseases like heart failure (Zhang et al., 2009; Wang and Zhao, 2018). Therefore, researchers have proposed “archaeabiotics” as the next generation of probiotics (Hania et al., 2017) to contribute to human health. Rumen archaea may have positive effects on livestock, with certain groups being significantly enriched in high-feed-utilization beef cattle, sheep rumen, and high-producing dairy cows. Li and colleagues found that archaea such as *M. smithii* are significantly enriched in the rumen of beef cattle with high feed efficiency (Li and Guan, 2017). Another study by Li et al. (2019) based on meta-transcriptomics, revealed that archaeal groups such as Methanomassiliicoccales exhibit heightened expression in the rumen of beef cattle with high feed efficiency. McLoughlin et al. (2020) identified three archaea associated with *Methanobrevibacter* spp. in the sheep rumen, and these archaea showed a significant positive correlation with feed efficiency. Xue et al.’s (2020) study identified the significant enrichment of 12 archaeal species in the rumen of high milk-yielding and high-milk-protein dairy cows. Feedback inhibition in fermentation by hydrogen gas arises from the accumulation of hydrogen, which in turn hinders the microbial activities generating it. These archaea help overcome the challenge of feedback inhibition of fermentation by hydrogen gas, maintaining a low hydrogen environment for efficient fermentation.

In pigs, archaea constitute a common component of the resident microbiota and actively participate in digestive and metabolic processes within the intestinal tract (Borrel et al., 2020). However, in comparison to ruminants, our understanding of the diversity of archaea in the pig gut, their potential functions, and their associations with economically significant traits remains limited. In this review, we summarize the current knowledge on pig gut archaea and their potential applications in the pig industry.

## 2. Diversity of gut archaea in pigs

Within the labyrinthine microbiome environment of the porcine digestive system, the Archaea—an extraordinary domain of life, distinct in phylogenetic, morphological, and physiological attributes—comprise a smaller fragment yet contribute significantly to microbial diversity. The genus *Methanocorpusculum* is particularly notable, and its relative abundance fluctuation is influenced by investigative parameters and sample diversity.

The 16S rRNA, found in bacterial and archaeal cells, possesses conserved sequences and is commonly employed as a marker gene (Liu et al., 2008). Trailblazing studies like those conducted by Mao et al. (2011) utilized 16S rRNA gene clone libraries to probe into the archaeal diversity present in pig fecal matter, revealing a dominance of *Methanobrevibacter* and *Methanosphaera stadtmanae* (Table 1). Concurrently, Luo and associates (Luo et al., 2012) emphasized the significant sequence homology between most archaeal sequences in Erhualian and Landrace pigs and *Methanobrevibacter*. Advancing beyond clone-based methodologies, Yong et al. (2014) employed DGGE (denaturing gradient gel electrophoresis) and RT-PCR (Reverse Transcription Polymerase Chain Reaction), illuminating *Methanosarcina* and *Methanobrevibacter* as the chief methane-producing archaea in fecal specimens. Their study also uncovered how porcine growth stages affect these archaea presences, along with *Methanosmithii* species. Yet, clone-based strategies might underrepresent archaeal diversity. Utilizing high-throughput sequencing technologies, Lamendella et al. (2011) and Su et al. (2014) revealed a diverse spectrum of methanogen-related operational taxonomic units (OTUs), with a significant majority aligning with the *Methanobacteria* class.

**Table 1:** Primary archaeal taxa in the pig gut.

Study number	Archaea taxa	Breeds	Age (day)	Technology	Citation
1	Methanobrevibacter (46%)	Duroc × Landrace × Yorkshire	Not given	16S rRNA sequencing	Borrel et al. (2020)
2	141 sequences (37%) related to *Methanobrevibacter gottschalkii* and *Methanobrevibacter millerae* 111 sequences (29%) related to *Methanobrevibacter smithii*	Erhualian × Landrace	40,50,70,330 ~ 360	16S rRNA sequencing	Liu et al. (2008)
4	*Methanomicrobia* and *Thermococci* account for 1% of the total rRNA sequences	Yorkshire	180	Metagenomic sequencing	Luo et al. (2012)
5	Across all reads, 99.91% were identified as class *Methanobacteria*	Meishan × Yorkshire	1,3,7,14	16S rRNA sequencing	Yong et al. (2014)
6	Euryarchaeota was the most abundance phylum Methanobacteriaceae, Methanosarcinaceae, and Candidatus Methanomethylophilaceae were the most abundant families *Methanobrevibacter smithii* was the most abundance species	Large White	14,21,28	Shotgun metagenomic sequencing	Quince et al. (2017)
7	*Methanobrevibacter* (23.02%) *Candidatus methanomethylophilus* (10.52%)	17 breeds from 11farms	Not given	Shotgun metagenomic sequencing	Sharpton (2014)
8	*Methanobrevibacter* A (0.23% of the total rRNA sequences) *Methanobrevibacter* (0.067% of the total rRNA sequences) *Methanomethylophilus* (0.062% of the total rRNA sequences) and *Methanosphaera* (0.023% of the total rRNA sequences)	Not given	7, 14, 21, 28, 35, 70, and 140	Shotgun metagenomic sequencing	Xiong et al. (2022)
10	Methanobacteriaceae family reaching 98.1% Methanomassiliicoccales reaching 1.8%	Landrace × Large White	28	16S rRNA sequencing	Deng et al. (2022)

### 2.1. Exploring archaea through metagenomics

Recently, shotgun metagenomic sequencing has gained traction due to its comprehensive and insightful data (Sharpton, 2014; Quince et al., 2017). Xiong et al. (2022) wielded this method to scrutinize gut archaea’s influence on bacterial colonization and succession in piglets, identifying Euryarchaeota as the predominant phylum and *Methanobrevibacter smithii* as the leading species. Deng et al.’s (2021) metagenomic examination of 276 samples corroborated these findings, naming *Methanobrevibacter* and *Methanobrevibacter A smithii* as the most copious archaeal genus and species, respectively. Deng et al.’s (2022) longitudinal analysis uncovered the temporal evolution of archaeal diversity in the swine gut, with weaning signifying a marked shift in community dynamics. The analysis revealed a significant positive correlation between the relative abundance of *Methanobrevibacter A sp900319535* and weight gain observed between days 70 and 140 (*p* = 0.005). Similarly, a potential positive correlation was noted between the relative abundance of *Methanobrevibacter A smithii* and weight gain (*p* = 0.005). These pivotal findings have implications for augmenting the economic efficacy of the livestock sector. Concurrently, Wei et al. (2022) elucidated the impact of domestication on archaeal abundance in the porcine gut, denoting a notable reduction in specific archaeal taxa.

### 2.2. Dynamic and differential characteristics of gut archaea

Research implies that archaea could significantly influence pig nutrition, metabolism, and growth performance, especially during weaning (Deng et al., 2022). The symbiotic relationship between methanogens and the host during the intestinal colonization of newborn piglets appears mutually selective (Su et al., 2014). Studies further note that gut fungal and archaeal communities exhibit high dynamism during lactation and weaning, with methanogens and parasites impeding piglets’ adaptation during the weaning transition (Xiong et al., 2022). Additionally, archeological traces have been found in various segments of the pig digestive tract, including the cecum, colon, rectum, and feces (Gresse et al., 2019). The dominance of the genus *Methanobrevibacter* was consistently observed, with the early colonization of methane-producing archaea in the intestines of piglets showing breed-specific variations and dramatic changes with age [18; 29]. Feehan et al. (2023) collected fecal samples from seven pigs at 22 time points for metagenomic sequencing. They found differences in the MAGs (Metagenome-Assembled Genomes) of the *Methanobacteriales* and *Methanomassiliicoccales* bacteria. The former predominantly existed in the gut of pre-weaning hosts, while the latter was mainly present in adult hosts, suggesting a potential relationship between specific groups of methanogenic archaea (Methanobacteriales and Methanomassiliicoccales) and the diet of the host at different life stages. Interestingly, the structure of archaeal communities in pig intestines seems heavily influenced by dietary patterns. Cao et al. (2016) provided Lantang pigs with varying fiber levels and documented significant alterations in the intestinal tract’s archaeal structure, notably *Methanobrevibacter*. Luo et al. (2012) also unveiled a higher diversity and density of archaea in fecal samples from lean-type pigs as compared to obese-type pigs, suggesting a possible role of intestinal archaea in pig fat deposition. These discoveries highlight the intricate, dynamic nature and potential roles of archaeal communities within the porcine gut, potentially influencing host health and productivity. Given the complexities presented, it is paramount to pursue further research endeavors that seek to delineate the intricate interplay between intestinal archaea, bacteria, and their respective hosts. Such investigations could potentially pave the way for innovative microbiota-centric interventions, thereby enhancing swine health and productivity.

## 3. Potential function of gut archaea in pigs

Archaea are crucial bacteria for ruminants. Numerous studies have focused on the function of archaea in the rumen. While certain archaeal taxonomies are beneficial for ruminants, most research suggests that rumen archaea, especially methanogenic archaea, reduce production efficiency (Huang et al., 2016; Kim et al., 2017; Huws et al., 2018; Xie et al., 2021). Even though the archaeal constituents within the pig gut microbiome may seem less significant in comparison to ruminants, it’s becoming increasingly clear that these microorganisms exhibit a unique dynamism and functionality. Peng et al. (2022) re-analyzed several datasets involved in metagenomic and meta-transcriptomic sequencing data of livestock, revealed a greater prevalence of ‘active archaeal species’ with a statistically significant *p*-adjust value of ≤0.05 and a fold change of ≥2, in pigs compared to sheep, cattle, and chicken. This finding suggests that archaea play crucial and active roles within the porcine gut, even when present in lower relative abundance compared with bacteria or fungi.

### 3.1. Potential role of gut archaea in fat metabolism

**Figure 1:**
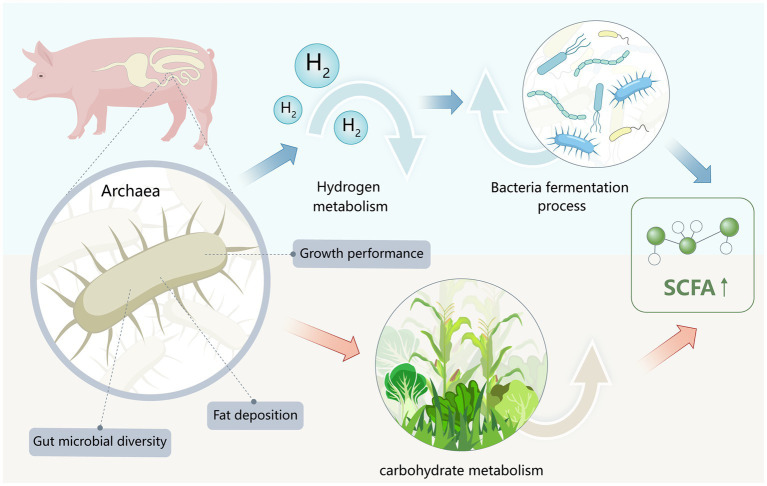
Potential functions and mechanisms of gut archaea in swine digestive systems.

The heightened presence of specific *Methanobrevibacter* species in the colonic environment of pigs, particularly when subjected to high-fat dietary conditions, has been noted. Zhao and colleagues collected samples of colonic contents from five high-fat pigs and low-fat pigs to explore the potential of link between gut microbiome and fat deposit (Zhao et al., 2022). Future studies with larger sample sizes are recommended to further elucidate the relationship between archaea and SCFA concentration, ensuring the results are more statistically significant and generalizable (Figure 1).

### 3.2. Impact of dietary composition on gut archaea in pigs

Dietary makeup significantly shapes the archaeal profile in pigs. For instance, levels of dietary fiber have been shown to adjust the abundance of *Methanobrevibacter* (Cao et al., 2016). Furthermore, researched by Luo et al. (2017) shows that the addition of dietary pea fiber boosts the diversity of methanogenic archaea and transforms the archaeal community structure. Lean and obese pigs display notable differences in the density and variety of fecal methanogens, implying a potential impact of archaea on fat deposition (Luo et al., 2012). In a study conducted by Xu et al. (2023), the increase of methane-inhibiting archaeal genera in experimental pig cohorts demonstrates the direct effect of dietary manipulation on gut archaeal diversity. However, the consequences of such shifts in the archaeal community on pig growth, development, and health call for further investigation.

### 3.3. Enhancing fermentation efficiency and growth performance

Our team’s scrutiny of the potential of pig gut archaea reveals their essential role in amplifying fermentation efficiency via hydrogen metabolism. A comprehensive examination of 276 pig fecal metagenomes discloses an exclusive hydrogen-consuming metabolic route in archaea, accompanied by a matching hydrogen-producing pathway in gut bacteria. This suggests a symbiotic interaction between archaea and bacteria, where archaea promote efficient gut fermentation by maintaining a low hydrogen environment (Deng et al., 2021). Additionally, the effects of weaning on the gut archaea of pigs demonstrate a positive correlation between archaeal species richness and pig body weight. The rise in functional potential, indicated by the increase in KEGG KOs over time, and the significant association of *Methanobrevibacter A smithii* and *Methanobrevibacter A sp900769095* with body weight, point toward archaea’s potential in enhancing pig performance (Deng et al., 2022). It is noteworthy to mention that the regression analysis utilized for these findings was based on a limited dataset of fewer than 25 samples, which may necessitate further studies for more comprehensive insights.

## 4. Potential application in the pig industry

Archaea, key components of the pig gut microbiome and primarily inhabitants of anaerobic environments, are central to host health and digestion. Despite receiving less attention than gut bacteria in research, current literature, inclusive of our findings, underscores their role in breaking down complex carbohydrates and producing vital short-chain fatty acids (SCFAs) (Samuel and Gordon, 2006). Our investigations suggest that enhancing *Methanosphaera* in pig diets can bolster feed efficiency (Deng et al., 2021). This endorses the manipulation of gut archaea as a promising approach toward sustainable pig farming. Moreover, archaea could serve as representatives of these traits. Associations have been noted between the abundance of certain archaea, specifically *Methanobrevibacter A smithii* and *Methanobrevibacter A sp900769095*, and traits like feed efficiency, growth rate, and meat quality (Lin and Miller, 1998; Deng et al., 2022; Zhao et al., 2022). Therefore, we propose that in the future, certain beneficial archaea could serve as “Archaebiotics” and play a crucial role in the swine industry. However, it’s important to recognize that this perspective is based on a few association studies. Further direct evidence is needed to firmly establish the influence of archaea on economically significant traits.

## 5. Outlook

Indeed, Archaea, often viewed as the linchpin species of the gut microbiome, orchestrate the functional establishment of bacterial communities within the host intestines. Their distinct metabolic capabilities and intimate interactions with bacteria regulate myriad vital processes connected to host health and growth. A more profound exploration of these intricate relationships could bring about transformational insights, shedding light on innovative strategies for bolstering animal health and productivity. However, unlike their extensively studied ruminant counterparts, pig gut archaea have been largely sidelined, obscuring their likely pivotal role in swine health and productivity.

Probiotic microorganisms, as living microbes, hold the capacity to regulate the equilibrium and dynamics of the gastrointestinal microbiota. Their constructive impacts on host health and productivity have led them to become one of the most favored additions to animal feed formulations (Retta et al., 2016; Markowiak and Śliżewska, 2018). The investigations by Peng et al. (2023) identified four strains of probiotics that exhibit potential inhibitory effects against pathogenic microorganisms. These inquiries highlight the pivotal role of probiotics in preserving the stability of the microbial community. Yet, of greater intrigue is the advent of ‘archaeal probiotics,’ which signals a pioneering phase in microbiome research. Initial indications hint at the potential of specific archaeal species to enhance host energy metabolism without inducing adverse effects (Luo et al., 2012), thus paving the way for their application as probiotics. Leveraging the strides in metagenomics and metabolomics could further illuminate the complex symbiotic relationships between archaea and their hosts. However, a significant challenge remains in the realm of archaeal research: the difficulty in culturing and isolating new archaeal species. This limitation not only hampers the comprehensive study of these microorganisms but also restricts their potential applications in various domains.

To summarize, our understanding of pig gut archaea is in its nascent stages, but existing evidence compellingly suggests the substantial roles these prokaryotes could play in swine health and productivity. As we progress, focused efforts should be aimed at better comprehending the ecological role of archaea, their interplay with other gut microbes, and their prospective benefits as probiotics. The untapped potential of gut archaea in pigs awaits our exploration.

## 6. Conclusion

This review has underscored the substantial yet understudied potential of pig gut archaea in the swine industry. The nascent understanding of the influence of archaea on pig nutrition, metabolism, and growth underscores their potential utility in augmenting swine health and productivity. The advent of sophisticated metagenomic studies, along with the isolation and functional verification of specific archaeal species, holds significant promise. This strategy may unearth the intricate roles these archaea play within pig gut ecosystems, leading to the development of innovative feed additives or probiotics. By encouraging further research into this area, we hope to fully exploit the beneficial aspects of these gut archaea and open up new avenues for sustainable and efficient pig farming.

## Author contributions

JY: Writing – original draft. RC: Writing – original draft. YP: Writing – review & editing. JC: Writing – review & editing. YL: Conceptualization, Writing – review & editing. FD: Conceptualization, Writing – original draft.
